# Evaluation of the safety and efficacy of a novel product for the removal of impacted human cerumen

**DOI:** 10.1186/s12901-017-0038-8

**Published:** 2017-06-02

**Authors:** Douglas Fullington, Jenny Song, Antionette Gilles, Xiaowen Guo, Waley Hua, C. Eric Anderson, Joseph Griffin

**Affiliations:** 1 Village Health Partners, 8080 Independence Pkwy, Plano, TX 75025 USA; 2 Health Cruiser Consulting LLC, 3504 Appalachian Ct, Plano, TX 75075 USA; 3 Wentian Huang MD, PhD PC, 3475 Collins Blvd, Garland, TX 75044 USA; 4 University of Texas, Southwestern Medical Center, 5909 Harry Hines Blvd, Dallas, TX 75235 USA; 5 Independent Medicinal Chemistry Consultant, 602 Nicholas Ct.,, Southlake, TX 76092 USA; 6 Eosera Inc., 1200 South Freeway Suite 132, Fort Worth, TX 76104 USA

**Keywords:** Cerumen removal, Cerumenolytic, Topical earwax removal

## Abstract

**Background:**

This open-label study evaluated the safety and efficacy of a novel product for the removal of impacted cerumen in adult patients.

**Methods:**

This was a prospective, single-center, single-arm, self-controlled clinical trial conducted in a community general practice setting. The novel product contains glycolic acid in an otologically-acceptable buffer containing sodium bicarbonate and glycerin and other buffering agents. The product was instilled into the ear canal prior to irrigation with warm water. Severity of cerumen impaction was graded using a 5-point scale. Improvement in tympanic membrane visualization was assessed after instillation and irrigation.

**Results:**

A majority (83%, 25/30) of ears showed improvement with one application: with 53% (16/30) totally dissolved and gained 100% TM visualization. Total dissolution was observed in 80% (24/30) of the study ears per the intent-to-treat analysis and 86% (24/28) if irrigation instructions were followed. Most of the ears/participants that had cerumen blockage symptoms experienced significant improvement with the application. Feelings of fullness disappeared in 92% (11/12) of the affected ears; ears itching, 91% (10/11); water trapping or cracking, 78%, and decreased hearing disappeared in 71% (10/14). All (100%, 18) of the participants who completed the application satisfaction assessment were satisfied with the application process in terms of time needed and the overall rinse procedure. Only one mild adverse event (ear pruritis) occurred that was related to application.

**Conclusions:**

The tested cerumen removal product was effective and safe for removing moderate to severe blockage in patients with impacted cerumen. Procedure satisfaction for the product was high.

**Trial Registration:**

This trial is registered on http://www.clinicaltrials.gov/. The registration number is NCT02829294. The trial was retrospectively registered on July 8, 2016.

## Background

Cerumen (earwax) serves to clean, lubricate, and protect the external auditory canal [1]. This substance is formed when glandular secretions from the outer portion of the canal mix with exfoliated squamous epithelium [2]. The migration of cerumen toward the periphery occurs similar to the rate of growth of a thumbnail. The movement of the jaw during chewing and talking promotes this migration [3]. This process represents a self-cleansing mechanism for the external auditory canal. Cerumen is generally classified into two types, the “dry” type, which is more prevalent in people of Asian descent and the “wet’ type, which is more prevalent among Caucasian and black individuals [3].

A number of factors cause individuals to be at an increased risk of cerumen impaction [1, 3–5]. Anatomical abnormalities may impede the natural extrusion of cerumen [3, 5].

Hair in the auditory canal may contribute to an increased risk of impaction [5]. Physical barriers to natural cerumen extrusion (e.g., cotton swabs, hearing aids, earplug-type hearing protectors) also can lead to impaction [1, 4, 5]. The accumulation of cerumen and eventual impaction is a common phenomenon.

Cerumen accumulation is one of the most common ear-related reasons for people to seek medical attention [6]. Excessive or impacted cerumen is estimated to be present in approximately 10% of children and 5% of adults [3]. As many as 65% of patients over the age of 65 years and up to 36% of those with mental retardation experience cerumen impactions [1, 3, 7, 8]. Even with these high proportions, excessive or impacted cerumen is likely underdiagnosed and undertreated [1]. Estimates from hearing aid manufacturers note that 60 to 70% of all hearing aids sent for repair are damaged because of contact with cerumen [1, 9]. Patients seek removal of cerumen impaction for a variety of symptoms including temporary hearing loss, ear pain, itching, a sensation of fullness, tinnitus, odor, drainage, and dizziness [1, 10]. In some cases, impacted cerumen can contribute to the development of otitis externa [3]. Cases of coughing or even cardiac depression have been associated with cerumen impaction [10–13].

Manual cerumen extraction is one of the most common ENT procedures performed in the primary care setting [10, 14]. Consequently, the healthcare burden of this condition is substantial [14]. About 12 million people in the United States seek medical attention for cerumen-based problems, resulting in almost 8 million ear irrigations being performed annually [1]. The removal of cerumen impactions can be painful and time consuming. General practitioners are becoming more reluctant to treat impactions due to complications associated with these interventions and often refer to ENTs for extraction [15, 16]. In addition, in many countries, including the US and the UK, audiologists’ extended scope of practice recently began to include cerumen management [17].

There are currently several cerumen products and methods used to address impactions. Products include water-based (e.g., acetic acid and triethanolamine), oil-based (e.g., almond oil, arachis oil, and camphor oil), and non-water, non-oil based (e.g., glycerol and propylene glycol) preparations [18]. Unfortunately, these agents tend to be minimally effective, often requiring multiple doses per day over several days to achieve satisfactory removal of cerumen [19, 20]. When patients try to remove cerumen themselves, they are unable to visualize the area they are cleaning. There can be a risk of damage to the tympanic membrane, particularly when vigorous water irrigation is used [1, 21]. Some products can cause allergic reactions in a small proportion of patients. Ear candling is generally not recommended since it is an ineffective and dangerous procedure [22–24] In fact, the United States Food and Drug Administration has issued a warning for consumers against the use of ear candles because they can cause serious injuries (such as perforations of the ear drum), even when used according to the manufacturer’s directions [23].

In light of these shortcomings, the need for a better cerumen removal product is apparent [25, 26]. This study evaluated the safety and efficacy of a novel product when applied topically in the ear canal of participants with moderate to severe impacted cerumen. The product is designed to be instilled into the ear canal prior to irrigation with warm water.

## Methods

### Study design

This was a single center (Legacy Medical Village, Plano, TX, USA), single-arm, open-label clinical trial where each patient served as his/her self-control. The study was registered on clinicaltrials.gov (NCT02829294). The patients were treated between April 17, 2016 and May 19, 2016.

The study included males or non-pregnant females ≥ 40 years of age at the time of enrollment. The participants were required to have at least 50% cerumen impaction in the ear canal to be enrolled.

Participants were excluded from the study if any of the following were present: a tympanostomy tube at any time during the previous 12 months, a non-intact tympanic membrane (TM), a known or suspected ear infection, known or suspected mastoiditis, pregnant or nursing mother, a condition or abnormality that in the opinion of the study investigator would compromise the safety of the patient or the quality of the data (e.g., ear eczema or seborrhea). The use of any ototopical drug or over-the-counter (OTC) product or cerumen-removal product (with the exception of water or physiologic saline) during the preceding 3 days was not allowed.

### Study visits

There were two scheduled study visits. Visit 1 involved participant screening and enrollment. At this time, the ears were assessed with an otoscope to determine if the tympanic membrane could be visualized. Percent area cerumen impacted around TM, depth impacted, and volumes impacted were recorded and the overall impaction was graded according to a 5-point disintegration scale (Table 1). This scale was adapted from those of Jimenez et al. [27] and Fraser et al. [28]. If both ears met the inclusion/exclusion criteria, both were included as individual ears in the study.

**Table 1:** Overall clinical score

1	<3%	Normal - Normal and/or insignificant earwax present in ear canal. Tympanic membrane completely visible.
2	3 – 25%	Minimal - Very little and mostly insignificant impacted cerumen that is not likely to have an effect on normal activities or cause any otologic or non-otologic symptoms; cerumen closer to the 25% impaction level may lead to increased impaction. Tympanic membrane (TM) is visible but still some minor presence of earwax.
3	26 – 50%	Mild - Some excessive impacted cerumen causing partial occlusion of the ear canal usually causing some minor to major otological and/or non-otological symptoms at the 30-50% level. TM partially visible, but somewhat difficult to see.
4	51 – 75%	Moderate - Moderate and excessive impacted cerumen causing partial occlusion of the ear canal causing major to serious complications in otological symptoms and in some cases, serious non-otological symptoms. Partial to very little of the TM visible.
5	76 – 100%	Severe - Severe and excessive impacted cerumen causing partial or *complete* occlusion of the ear canal; these subjects may have significant qualify of life issues with the complications from the otological and non-otological symptoms. Little if any of the TM is visible.

At Visit 2, participants were re-evaluated for eligibility. At this time, the participants received application of the test eardrop. Prior to and following lavage, otoscopic examinations were performed. Participants then provided subjective assessments without knowing the outcome of the otoscopic exams (visibility of the TM). When visualization of the TM was not possible, or the ear canal was not clinically clear of the cerumen (with exception of normal occurring cerumen that was less than 3%), the application process was repeated a second time. Following the completion of the ear canal evaluation, the subjective procedure satisfaction questions were posed.

### Test product

The test product has been designed to take advantage of the chemical characteristics of the various components of human cerumen, in particular the lipid/wax component, and the un-separated keratinocytes. Ingredients were identified that could quickly, safely, and effectively dissolve human cerumen when combined. The new liquid product uses a ‘dual-action’ mechanism to dissolve human cerumen. The bicarbonate system disrupts the wax ester and fatty acid lipid components of the cerumen [25, 26]. By targeting the ester linkage and the carboxylic acid linkage, it breaks these molecules down to their corresponding carboxylate salts, which are much more water-soluble. The glycolic acid system acts to sequester (chelate) calcium ions from the calcium-dependent cell adhesion molecules causing disruption of cadhedrins allowing the keratin sheet cells to break apart [29, 30].

### Application procedures

All ears received at least a single application (instillation of approximately 1 mL) of the test product applied topically in the study ear canal by the treating physician. The study participant was dosed with the head tilted in order to keep the test product in the ear canal for 15 min. At 5 and 10 min, participants were instructed to move their jaw up and down (and side to side) a few times and manipulate/massage the ear canal by pressing between jawbone and ear lobe with a rotating motion for 10 s. Since jaw movement promotes the migration of cerumen outward toward the opening of the ear canal [3], these manipulations were expected to aid in distribution of the product in the ear canal. The product was removed 15 min after instillation by having the participant tilt the head over a disposable container to collect the solution and cerumen. The ear canal was then irrigated (‘rinsed’ under low pressure) with warm water using an ear syringe. If a second application was needed, it was performed immediately after the first application. The test product was supplied from a qualified compounding pharmacy and stored at temperatures from 59 °F to 86 °F.

### Symptom assessments

Participants were asked to provide a self-assessment of otological symptoms related to cerumen impaction before and after the application. The otological symptoms collected with cerumen impaction during this study included the participant’s perception of hearing loss, aural fullness, cracking (with or without water exposure), tinnitus, itching/pruritus, and ear discomfort. Participant’s perceptions of dizziness, restlessness, anxiety, and impact on their overall quality of life (before and after application) were recorded.

### Procedure satisfactory survey

Subjective assessments included the overall assessment of improvement and test product acceptability. Participants were asked if they thought the test product worked on reducing the cerumen impaction (Yes, No, No Opinion, or NA response). The following questions were asked:

1. Were you satisfied with the application process in terms of the time you kept your head to the side with it in your ear?
2. Were you satisfied with the overall comfort or feeling of the solution in your ear?
3. Did the warm water rinse bother you as part of the application process?
4. Were you satisfied with the application process including the warm water rinse?

### Adverse events

Adverse events (AEs), both serious and nonserious, were recorded. Relationships of AEs to the test product were determined by the physician as unrelated, possibly, probably or definitely related to the testing product.

### Statistical analyses

All participants who received the test product were included in the safety and efficacy analyses. The primary efficacy variable was the improvement in visualization of the tympanic membrane following application when compared to pre-application. Standard descriptive statistics are presented for ear canal symptoms and application satisfaction. For within-subject/ear before-after application comparisons, Pearson’s Chi-square test was used. For dichotomized variables, McNemar test was used. A 95% confidence level was used for all tests. Statistical analyses were conducted using Statistical Analysis Software -PC 9.4 (SAS Institute, Cary, NC) by an independent biostatistician.

Sample size was determined based on the improvement in TM visualization that a study with 30 evaluable ears would have at least 80% statistical power at 95% confidence level to detect an improvement of at least 20% in TM visualization compared to pre-application.

## Results

### Demographics

A total of 35 ears of 24 patients were screened for impacted cerumen. Of these, 5 ears of 5 patients were disqualified according to the study inclusion/exclusion criteria. This resulted in 30 ears in 19 participants being enrolled into the study. Eight of the participants had one ear enrolled while 11 participants had both ears enrolled. Most of the study participants were males (79%) and the average patient age was 64.8 years (±12.3 standard deviation, SD).

### Baseline disease characteristics

The most frequent complaint related to cerumen impaction at the baseline assessment was decreased hearing (50%), followed by a sensation of fullness (40%), and ringing in the ear (40%) (Table 2).

**Table 2:** Baseline cerumen blockage symptoms – Ear specific symptoms

Variable	Yes *n* (%)	No *n* (%)
Decreased hearing	15 (50)	15 (50)
Feeling of fullness	12 (40)	18 (60)
Ringing or noises in the ear (tinnitus)	12 (40)	18 (60)
Ear itching	11 (37)	19 (63)
Water trapping or cracking noise after swimming or shower	10 (33)	20 (67)
Ear irritation/discomfort	3 (10)	27 (90)
Earache, tingling or pain	1 (3)	29 (97)
Total = 30 ears from 19 participants		

Visit 1 otoscopic evaluation

In the cerumen evaluation immediately prior to application, 67% of ears had severe ear impactions (76% to 100% TM visualized, Table 3). Half of the occlusions (50%) were classified as fully occluded, while 17% of the occlusions were ring shaped and another 33% were crescent shaped. Most (80%) of the occlusions were classified as being wet with a normal consistency.

**Table 3:** Cerumen evaluation at enrollment

Variable	# of Ears	Percent (%)
Ear Impaction		
51–75% (moderate)	10	33
76–100% (severe)	20	67
Shape		
Full occlusion	15	50
Ring	5	17
Crescent	10	33
Appearance		
Wet, normal	24	80.00
Wet, tarry	0	---
Wet, firm nuggets	0	---
Dry, normal	4	13
Dry, flakes	0	---
Dry, packed	2	7
Total	30	100

Seven out of the 19 (37%) participants reported that cerumen impaction had negatively impacted their overall quality of life (data not shown). Only two (11%) complained of dizziness, while none complained of restlessness or anxiety. Eleven (58%) participants did not have any systemic symptoms and 7 (37%) complained of at least one (dizziness or an impact on overall quality of life), while only one participant had two.

The presence of cerumen blockage symptoms was not associated with the severity of impaction (*P* ≥ 0.2451). Otoscopic examinations immediately prior to the 1st instillation indicated that there were 18 (60%) and 12 (40%) ears, respectively, that had severe and moderate blockage.

### Application efficacy

Fifteen (15) minutes after the instillation of the product, the ears were drained by tilting the head downward. Blockage in two (17%) of the moderate ears had reduced to minimal (3 to 25% blockage) and 4 (33%) to mild (26 to 50% blockage) (Table 4). Improvement to moderate blockage was observed in 5 (28%) of the severe ears.

**Table 4:** Application evaluation – 1st instillation

	Impaction before 1st instillation *n* (%)		
Irrigation	51-75%	76-100%	Total
Ear impaction *before* 1st irrigation			
<3% impacted	0	0	0
3–25%	2 (17)	0	2 (7)
26–50%	4 (33)	0	4 (13)
51–75%	6 (50)	5 (28)	11 (37)
76–100%	0	13 (72)	13 (43)
Ear impaction *after* 1st irrigation			
<3% impacted	4 (33)	3 (17)	7 (23)
3–25%	2 (17)	1 (6)	3 (10)
26–50%	4 (33)	2 (11)	6 (20)
51–75%	2 (17)	2 (11)	4 (13)
76–100%	0	10 (56)	10 (33)
Ear impaction *after* 2nd irrigation			
<3% impacted	8 (67)	3 (17)	11 (37)
3–25%	0	2 (11)	2 (7)
26–50%	3 (25)	2 (11)	5 (17)
51–75%	1 (8)	3 (17)	4 (13)
76–100%	0	8 (44)	8 (27)
Ear impaction *after* 3rd irrigation			
<3% impacted	8 (67)	8 (44)	16 (53)
3–25%	0	1 (6)	1 (3)
26–50%	3 (25)	2 (11)	5 (17)
51–75%	0	2 (11)	2 (7)
76–100%	0	4 (22)	4 (13)
Not performed	1 (8)	1 (6)	2 (7)
2nd treatment needed			
No	8 (67)	8 (44)	16 (53)
Yes	4 (33)	10 (56)	14 (47)
Total	12 (100)	18 (100)	30 (100)

After the first irrigation, with one small bulb of warm water, cerumen in 7 (23%) of the ears, including 4 (33%) moderate and 3 (17%) severe ears, was totally dissolved. Minimal or mild blockage was observed in 6 (50%) moderate and 3 (17%) severe ears. Improvement to moderate blockage was observed in 2 (11%) severe ears.

A second irrigation was applied to 23 ears that had at least minimal blockage. Cerumen in another 4 of the moderate ears was totally dissolved, resulting in a total of 11 (37%) ears with total dissolution after two rinses. One (8%) moderate and 8 (44%) severe ears had not shown obvious improvement in TM visualization.

A third irrigation was applied to 17 ears. The 3rd irrigation totally dissolved the cerumen in another 5 (28%) severe ears, resulting in a total of 16 (53%) ears with total dissolution at the end of the 1st application. Two participants elected to have a 2nd application instead of having a third rinse. With the exception of 5 ears with severe blockage, a majority (83%) had substantial reduction in cerumen after 1 application: totally dissolved and gained 100% TM visualization (53%), partially dissolved to minimal (3%) or mild (20%), or reducing blockage from severe to moderate (7%).

As expected, the cerumen from patients with full occlusions was more difficult to dissolve. Only 13% (3 of 15) of the cerumen occlusions dissolved partially without rinsing, compared to 80% (4 of 5, 2 of them totally dissolved) of those with ring-shaped and 70% (7 of 10) with crescent-shaped cerumen occlusions.

A second application was applied to 14 ears that had not been totally dissolved at the conclusion of the 1st application. One participant with bilateral cerumen occlusion elected to have physical removal instead of irrigations. Therefore, 13 ears underwent irrigation. With one rinse, 4 (31%) additional ears were totally cleared, one improved from mild to minimal, and one from severe to moderate, while the remaining 7 (53%) showed no obvious change from their severity since before irrigation (Table 5).

**Table 5:** Application Evaluation – 2nd Instillation

	Impaction before 2nd Instillation *n* (%)				
Irrigation [*n* (%)]	3–25%	26–50%	51–75%	76–100%	Total
Ear impaction *before* 1st irrigation					
<3% impacted	0	0	0	0	0 (0)
3–25%	1 (100)	1 (17)	0	0	2 (14)
26–50%	0	5 (83)	0	0	5 (36)
51–75%	0	0	2 (100)	0	2 (14)
76–100%	0	0	0	5 (100)	5 (36)
Ear impaction *after* 1st irrigation					
<3% impacted	1 (100	2 (33)	0	1 (25)	4 (31)
3–25%	0	1 (17)	0	0	1 (8)
26–50%	0	3 (50)	0	0	3 (23)
51–75%	0	0	2 (100)	1 (25)	3 (23)
76–100%	0	0	0	2 (50)	2 (15)
Not performed^a^	0	0	0	1 (--)	1 (--)
Ear impaction *after* 2nd irrigation					
<3% impacted	1 (100	2 (33)	0	3 (75)	6 (50)
3–25%	0	3 (50)	0	0	3 (25)
26–50%	0	1 (17)	0	0	1 (8)
51–75%	0	0	1 (100)	0	1 (8)
76–100%	0	0	0	1 (25)	1 (8)
Not performed^a^	0	0	1 (--)	1 (--)	2 (--)
Ear impaction *after* 3rd irrigation					
<3% impacted	1 (100)	4 (67)	0	3 (75)	8 (67)
3–25%	0	1 (17)	0	0	1 (8)
26–50%	0	1 (17)	1 (100)	0	2 (17)
51–5%	0	0	0	0	0 (0)
76–100%	0	0	0	1 (25)	1 (8)
Not performed^a^	0	0	1 (--)	1 (--)	2 (--)
Physical removal necessary					
No	1 (100)	5 (83)	0	3 (60)	9 (64)
Yes	0	1 (17)	2 (100)	2 (40)	5 (36)
Total	1 (100)	6 (100)	2 (100)	5 (100)	14 (100)

^a^Not performed due to subject refusal. Removed from the irrigation-specific analysis

Eight ears underwent a 2nd irrigation. Of these, two ears with severe impaction reached total dissolution while two improved from mild to minimal. Six ears underwent a 3rd irrigation. Two ears with minimal blockage after the 2nd irrigation reached total dissolution, one improved from moderate to mild, and the remaining 3 ears showed no obvious improvement.

At the conclusion of the 2nd application, of the 28 ears that completed the per-protocol application, 24 (86%) ears reached total dissolution, 4 (11%) showed cerumen impactions that were partially dissolved and one (4%) showed no obvious improvement from baseline in terms of TM visualization (data not shown). Overall, with all study ears accounted for, including the two protocol deviations (missing irrigations), the total dissolution rate was 80%.

Physical removal with a curette was performed on 5 (17%) ears after the 2nd application. The cerumen was noticeably softened and easy to remove with a curette in all these cases. Four of these 5 ears had full occlusions at baseline, 3 appeared wet and normal and the other was dry and packed at baseline. Another ear had moderate impaction in a crescent shape with wet and normal appearance. The correlation analysis did not detect any statistically significant correlation between baseline cerumen characteristics and the need for physical removal. However, the subgroups were too small to draw clinically meaningful conclusions.

### Symptom improvement

**Fig. 1:**
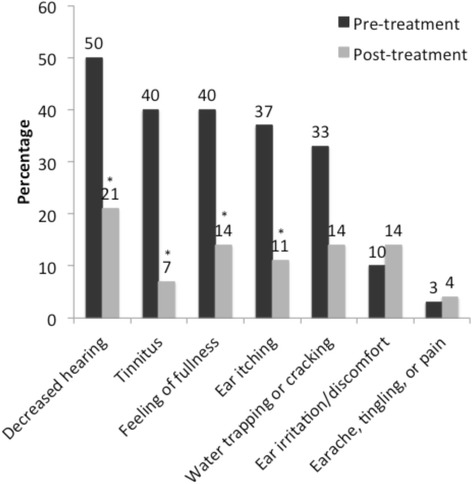
Cerumen blockage symptoms before and after application with the cerumen-removal product. Pre-application, *n* = 30 ears. Post-application, *n* = 28 ears. Significant improvements were observed after application for decreased hearing (*P* = 0.0209), tinnitus (*P* = 0.0027), feelings of fullness (*P* = 0.0325), and ear itching (*P* = 0.0209)

Most of the participants that had cerumen blockage symptoms experienced substantial improvement with application. A high proportion of patients experienced disappearance (improvements) of these blockage symptoms in the affected ears (feelings of fullness in 92%; ears itching in 91%; water trapping or cracking in 78%, and decreased hearing in 71%) (data not shown). Significant improvements were observed after application for decreased hearing (*P* = 0.0209 per McNemar test), tinnitus (*P* = 0.0027), feelings of fullness (*P* = 0.0325), and ear itching (*P* = 0.0209) (Fig. 1). After the application, a few participants indicated that they had experienced “new” symptoms that they did not have prior to application such as feeling of fullness (3 ears), ear irritation or discomfort (3 ears). Study participants experienced significant improvements in their quality of life after application (*P* = 0.0253).

**Fig. 2:**
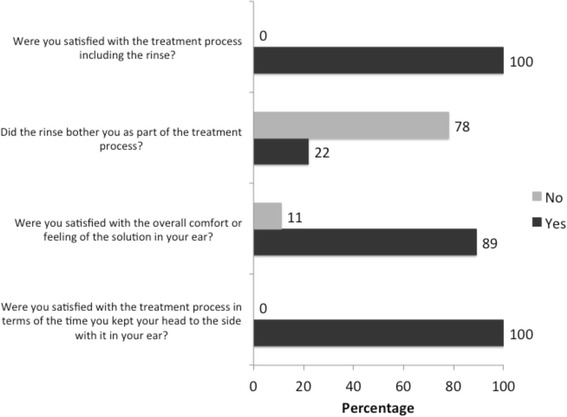
Application satisfaction. *n* = 18 participants. The application satisfaction assessment was not available for one participant

Eighteen (95%) out of 19 participants completed the Application Satisfactory Assessment (Fig. 2). All of them were satisfied with the application process in terms of the time they had to keep their head tilted to the side with solution inside the ear and the application process including the rinse.

### Adverse events

Only one patient complained of mild pruritis (itching) of the ear as a result of the application. This patient had a moderate blockage in one ear with every ear-specific blockage symptom present. The blockage was totally cleared with one instillation and two rinses. In the post-application survey, this patient expressed that the rinses bothered, and indicated that all symptoms, including ear itching, were absent except water trapping. The event resolved without any treatment. The other four events, all mild in nature, were considered unrelated to application (Table 6).

**Table 6:** List of adverse events

Subject ID	Ear	Description	Severity	Relation to treatment
102	Right	Irrigation taking earwax off tissue, no bleeding	Mild	Not related
104	Left	Water from flushing, one droplet on TM	Mild	Not related
	Right	Water from flushing, one droplet on TM	Mild	Not related
112	Right	Right ear discomfort	Mild	Not related
114	Right	Pruritis of right ear	Mild	Related

Total = 5 events for 5 ears from 4 subjects

## Discussion

The current study demonstrated safety and efficacy of the new cerumen removal product. A majority of ears with moderate impactions at baseline (83%) showed improvement with one application (e.g., 1 application and irrigation). Current OTC cerumen removal products/methods have some disadvantages [1]. Studies have indicated they are less effective or no better than deionized water [20, 31]. The current OTC products clear cerumen less than half of the time [6, 18]. Systemic reviews have found no topical cerumenolytic clearly superior to any other or to saline or sterile water [18, 32, 33]. They often require multiple doses per day over the course of several days.

The current study demonstrated the effectiveness of the test eardrop for application of moderately to severely impacted cerumen. With a single 15-min application regimen, about half of the ears (53%; 16/30) achieved cerumen that was totally dissolved with 100% TM visualization. A total of 83% showed improvement. With a second application regimen, the total dissolution rate increased to 80% and a total of 93% showed improvement. When the irrigation instructions were strictly followed, the total dissolution rate increased to 86% (24/28). This degree of efficacy is noteworthy compared with the currently available products, which often required multiple administrations over multiple days in order to dissolve or remove cerumen impactions. Four of the five ears that underwent physical removal with a curette had full occlusions at baseline. The cerumen in these ears was noticeably softened and easy to remove following two applications.

It was not surprising to observe that a second application may have been related to the shape of the impaction. All five ring shaped impactions (3 moderate and 2 severe) were totally dissolved with one application. Ears needing the second application had full or crescent-shaped occlusions. The ears with full occlusions likely had more buildup of cerumen than those that were partially occluded. These results suggest early intervention with the cerumen removal product (e.g., before the ear becomes completely occluded) would be beneficial.

A previous study compared the new product with two commercially available products, which both contained carbamide peroxide 6.5%, for their efficacy as cerumenolytic agents in vitro [34]. The cerumen samples exposed to the product containing sodium bicarbonate, glycerin, and other buffering agents demonstrated significantly greater disintegration than the carbamide peroxide products at all the time points examined (5, 10, 15, and 30 min). Moreover, the cerumenolytic activity of new product was observed within 5 min. Although the current study was not comparative in nature, these in vitro results suggest the further controlled comparative clinical studies with the new formulation and other available treatments are warranted.

Other studies have evaluated the efficacy of different cerumenolytic formulations. Oron et al. [35] tested three products: one containing carbamide peroxide and anhydrous glycerin; another containing mineral oil, squalene and spearmint oil; and the third containing peanut oil, chlorobutanol, and dichlorobenzene. The study evaluated the efficacy of treatment performed 3 times a day for 1 week. Resolution of ear occlusion was achieved in 38 to 54% of the ears that received treatment. No differences were found between the 3 products in terms of the degree of obstruction following treatment. Another study by Roland et al. [19] compared the efficacy of 3 cerumenolytic agents. The study protocol allowed up to two 15-min treatments followed by irrigation. The resolution of cerumen occlusion for the group receiving 10% triethanolamine polypeptide oleate condensate was 29.2%. The group treated with 6.5% carbamide peroxide achieved a resolution of 15.4% while the saline placebo group achieved 41.7% resolution. No significant differences were found between the 2 test agents and the placebo. The current study results compare favorably with these previous studies as participants achieved 80% to 86% resolution of occlusion with only two applications.

A significant proportion of participants experienced relief of symptoms associated with cerumen impaction such as the feeling of fullness, ear itching, tinnitus, and temporary reductions in hearing. Application also significantly improved the overall quality of life as experienced by the study participants. These results also support prophylactic or maintenance administration of the cerumen-removal product so that accumulations that could cause temporary losses in hearing and negatively affect an individual’s quality of life would be prevented. Adverse events were few and none were severe. Eighty percent (4 out of 5) of the adverse events were deemed not related to application. Mild pruritis of the ear was the only event thought by the investigator to be application related.

The satisfactory rate relating to the application process was 100%. Participants were generally satisfied with the application process in terms of time involved, the comfort of the application process, and the rinsing procedure.

Manual cerumen extraction is one of the most common ENT procedures performed in primary care [10, 14]. In 2012, the Centers for Medicare & Medicaid Services reimbursed $46.8 million for 1.3 million cerumen disimpactions [14]. Even with the large number, the condition is probably underdiagnosed. Elderly patients may have difficulty differentiating the buildup of cerumen with the natural decline in hearing acuity. The percentage of beneficiaries receiving cerumen extractions per state has been shown to range from 0.55 to 4.92% [14]. These procedures are performed by otolaryngology-head and neck practitioners (67.60%), internal medicine (32.66%) and family practice (33.87%) doctors [14]. In addition, the Academy of Doctors of Audiology lists cerumen management as a service that audiologists provide [15]. Manual cerumen removal can be time consuming for both the patient and the health care provider, creating a burden on health care resources that might be better utilized for more serious conditions. Cerumen is a product produced by the body to service the unique anatomical characteristics of the external ear canal [10]. Due to the cul-de-sac design of the outer ear canal, normal physical epithelial erosion (seen with the majority of bodily skin cells) does not occur.

Cerumen assists in the removal of ear canal epithelial cells (corneocytes/keratinocytes). The uniquely modified secretory glands contained in the outer ear canal (e.g., sebaceous and ceruminous glands) secrete long chain fatty acids/lipids and wax esters into the ear canal, along with secretions from hair follicles located within the canal. These secretions serve to lubricate the ear canal to help keep the ear clean. In some cases of excess or impacted cerumen, histological reports in the literature show long sheets of undivided keratin cells contained within plugs of cerumen [36]. Keratin comprises much as 60% of the contents of the cerumen plug [37]. These undivided keratin sheets are essentially epithelial cells that have maintained cell-to-cell adhesions (facilitated by cadhedrans and other cell adhesion molecules) and have not completed full desquamation. The glandular secretions combine with the un-separated keratinocyte sheets to create a cerumen plug. Over time, this plug can expand to a size that can no longer be removed by the natural clearing process.

The current exploratory study had some limitations, which should be mentioned. This study was open label, had a small sample size, and was conducted at only one center. Due to the single arm design, there was no reference product for which to make comparisons. However, these shortcomings can be addressed in a larger comparative multicenter randomized masked clinical trial.

## Conclusions

This new dual-action product addresses a need for a more effective agent to remove cerumen impactions. This dual-action mechanism is the key difference between this new cerumen-removal product and other over-the-counter products that are currently available. Intuitively speaking, if people use it as maintenance/routine hygiene product, before the onset of symptoms or before symptoms became too severe, the likelihood of total dissolution could be very high. As a result, cerumen related hearing loss and symptoms could be prevented which will improve overall quality of life. This could reduce or eliminate the need for intervention from a physician. In turn, related health care resources due to effective cerumen removal in one application, costs would be greatly reduced.

## Abbreviations

AEs: Adverse Events
ENT: Ear, nose and throat physicians
OTC: Over-the-counter
SD: Standard deviation
TM: Tympanic membrane
UK: United Kingdom
US: United States

## References

[1] Roland PS, Smith TL, Schwartz SR, Rosenfeld RM, Ballachanda B, Earll JM (2008). Clinical practice guideline: cerumen impaction. Otolaryngol Head Neck Surg.

[2] Alberti PW (1964). Epithelial migration on the tympanic membrane. J Laryngol Otol.

[3] Roeser RJ, Ballachanda BB (1997). Physiology, pathophysiology, and anthropology/epidemiology of human earcanal secretions. J Am Acad Audiol.

[4] Lewis-Cullinan C, Janken JK (1990). Effect of cerumen removal on the hearing ability of geriatric patients. J Adv Nurs.

[5] Meador JA (1995). Cerumen impaction in the elderly. J Gerontol Nurs.

[6] McCarter DF, Courtney AU, Pollart SM (2007). Cerumen impaction. Am Fam Physician.

[7] Garahan MB, Waller JA, Houghton M, Tisdale WA, Runge CF (1992). Hearing loss prevalence and management in nursing home residents. J Am Geriatr Soc.

[8] Moore AM, Voytas J, Kowalski D, Maddens M (2002). Cerumen, hearing, and cognition in the elderly. J Am Med Dir Assoc.

[9] Kochkin S (2005). Customer satisfaction with hearing instruments in the digital age. Hear J.

[10] Guest JF, Greener MJ, Robinson AC, Smith AF (2004). Impacted cerumen: composition, production, epidemiology and management. QJM.

[11] Raman R (1986). Impacted ear wax--a cause for unexplained cough?. Arch Otolaryngol Head Neck Surg.

[12] Prasad KS (1984). Cardiac depression on syringing the ear. A case report. J Laryngol Otol.

[13] Paulose KO, Shenoy PK, Sharma RK (1988). Otogenic reflex cough: implanted hair in the bony external auditory canal. Arch Otolaryngol Head Neck Surg.

[14] Yang EL, Macy TM, Wang KH, Durr ML (2016). Economic and demographic characteristics of cerumen extraction claims to Medicare. JAMA Otolaryngol Head Neck Surg.

[15] Pothier DD, Hall C, Gillett S (2006). A comparison of endoscopic and microscopic removal of wax: a randomised clinical trial. Clin Otolaryngol.

[16] Manchaiah V, Arthur J, Williams H (2015). Does hearing aid use increase the likelihood of cerumen impaction. J Audiol Otol.

[17] Wilson PL, Roeser RJ (1997). Cerumen management: professional issues and techniques. J Am Acad Audiol.

[18] Hand C, Harvey I (2004). The effectiveness of topical preparations for the treatment of earwax: a systematic review. Br J Gen Pract.

[19] Roland PS, Eaton DA, Gross RD, Wall GM, Conroy PJ, Garadi R (2004). Randomized, placebo-controlled evaluation of Cerumenex and Murine earwax removal products. Arch Otolaryngol Head Neck Surg.

[20] Rojahn R (2010). Summaries of nursing care-related systematic reviews from the Cochrane Library: Ear drops for the removal of ear wax. Int J Evid Based Healthc.

[21] Schmiemann G, Kruschinski C (2009). Complication rate of out-patient removal of ear wax: systematic review of the literature. HNO.

[22] Zackaria M, Aymat A (2009). Ear candling: a case report. Eur J Gen Pract.

[23] Hornibrook J (2012). Where there’s smoke there's fire--ear candling in a 4-year-old girl. N Z Med J.

[24] Rafferty J, Tsikoudas A, Davis BC (2007). Ear candling: should general practitioners recommend it?. Can Fam Physician.

[25] Bortz JT, Wertz PW, Downing DT (1990). Composition of cerumen lipids. J Am Acad Dermatol.

[26] Carr MM, Smith RL (2001). Ceruminolytic efficacy in adults versus children. J Otolaryngol.

[27] Jimenez N, Garcia ML, Galan J, Vallet A, Owen GR, Wall GM (2008). Development of a liquid enzyme-based ceruminolytic product. J Pharm Sci.

[28] Fraser JG (1970). The efficacy of wax solvents: in vitro studies and a clinical trial. J Laryngol Otol.

[29] Kornhauser A, Coelho SG, Hearing VJ (2010). Applications of hydroxy acids: classification, mechanisms, and photoactivity. Clin Cosmet Investig Dermatol.

[30] Wang X (1999). A theory for the mechanism of action of the alpha-hydroxy acids applied to the skin. Med Hypotheses.

[31] Saxby C, Williams R, Hickey S (2013). Finding the most effective cerumenolytic. J Laryngol Otol.

[32] Burton MJ, Doree C. Ear drops for the removal of ear wax. Cochrane Database Syst Rev. 2009;(1):CD004326. 10.1002/14651858.CD004326.pub2 19160236

[33] Burton MJ, Doree CJ. Ear drops for the removal of ear wax. Cochrane Database Syst Rev. 2003;(3):CD004400.10.1002/14651858.CD00440012918014

[34] Knebl J, Harty B, Anderson CE, Dean WD, Griffin J. In vitro comparison of three earwax removal formulations for the disintegration of earwax Submitted. 2017

[35] Oron Y, Zwecker-Lazar I, Levy D, Kreitler S, Roth Y (2011). Cerumen removal: comparison of cerumenolytic agents and effect on cognition among the elderly. Arch Gerontol Geriatr.

[36] Robinson AC, Hawke M, MacKay A, Ekem JK, Stratis M (1989). The mechanism of ceruminolysis. J Otolaryngol.

[37] Robinson AC, Hawke M, Naiberg J (1990). Impacted cerumen: a disorder of keratinocyte separation in the superficial external ear canal?. J Otolaryngol.

